# Lethal Male Combat of *Anastatus japonicus* (Hymenoptera: Eupelmidae), an Egg Parasitoid of Lepidopterous and Hemipterous Pests

**DOI:** 10.3390/insects16010045

**Published:** 2025-01-06

**Authors:** Muhammad Yasir Ali, Gonzalo A. Avila, Zheng-Yu Luo, Muhammad Asghar Hassan, Khalid Ali Khan, Jin-Ping Zhang, Feng Zhang

**Affiliations:** 1 MARA-CABI Joint Laboratory for Bio-Safety, Institute of Plant Protection, Chinese Academy of Agricultural Sciences, Beijing 100193, China; y.ali@cabi.org (M.Y.A.); luozhenggyu@163.com (Z.-Y.L.); 2 Centre for Agriculture and Biosciences International (CABI), Beijing 100081, China; 3 The New Zealand Institute for Plant and Food Research Limited, Auckland Mail Centre, Auckland 1025, New Zealand; gonzalo.avila@plantandfood.co.nz; 4 The Provincial Special Key Laboratory for Development and Utilization of Insect Resources, Institute of Entomology, Guizhou University, Guiyang 550025, China; kakojan112@gmail.com; 5 Center of Bee Research and Its Products (CBRP), and Research Center for Advanced Materials Science (RCAMS), King Khalid University, P.O. Box 9004, Abha 61413, Saudi Arabia; kkhan@kku.edu.sa; 6 Applied College, King Khalid University, P.O. Box 9004, Abha 61413, Saudi Arabia

**Keywords:** male parasitoid, aggression, body size, local mate competition, food, male relatedness

## Abstract

Aggressive interactions between males are common when victors gain increased mating success but can result in severe injury or death for the defeated. *Anastatus japonicus* (Hymenoptera: Eupelmidae) is a solitary egg parasitoid of hemipteran and lepidopteran species. Here, we investigated lethal interactions between *A. japonicus* males and analyzed aggression behavior scaled with the male condition, number of competitors, number of presented females, and female mating status. The intensity of *A. japonicus* male aggressiveness increased with the number of competitors and the number of presented females but was not affected by male relatedness. The intensity and frequency of *A. japonicus* male aggressive interactions were greater when males competed for virgin females compared to mated females. Larger *A. japonicus* males won more contests than smaller males and honey-fed males defeated starved ones, where defeated males suffered the loss of appendages and/or died. Fighting appeared to be the norm in all-male groupings, regardless of the presence of females. A number of biological and ecological factors are deducted that likely contribute to the evolution of male aggressive behavior in *A. japonicus*.

## Simple Summary

*Anastatus japonicus* is an effective egg parasitoid of lepidopterous and hemipterous pests; however, potential male–male combat happens when critical resources become limited. The greatest aggressive interactions between *A. japonicus* males were found to occur over virgin females. The outcomes of the fights were not affected by male relatedness, rejecting Hamilton’s theory of kin selection. Larger males won fight contests over smaller males, and honey-fed males defeated starved ones, resulting in the loss of appendages and/or death. We explored possible variables that may have contributed to the evolution of lethal conflict in this species by combining our discussion with the biological characteristics of *A. japonicus*.

## 1. Introduction

In the animal world, competition for mates can select for aggressive behavior toward same-sex conspecifics, usually males. Contests generally occur when a critical resource (females, in the case of male–male competition) becomes limiting, such that the advantages of defeating the adversary (mating success) exceed the potential costs of conflict (injury, dismemberment, death) [1]. When females mate only once, as in many arthropod species, this reduces mating opportunities for males and precludes the possibility of any post-copulatory mechanisms for improving paternity (e.g., sperm competition, sperm removal, mating plugs, etc.) [2,3].

Lethal contests for mates occur when females mate only once or limited times and have a short reproductive lifespan [4]. Contests for mating have been well studied in several insect species, including *Cardiocondyla* ants, to understand how long young males are vulnerable and how early adult males can detect the presence of emerging rivals in their nests [5]. In addition, *Philotrypesis pilosa* fig wasps’ mandible lengths predict resource-holding potential (RHP) and quantify the relationship with the probability of winning a fight [6,7]. Fighting in *Melittobia* species was tested for the importance of competitors and kin [8]. Furthermore, in these insects, the occurrence and intensity of contests are influenced by the value of the resource, relatedness, and competitor density [9].

The cost–benefit ratio of fighting in animals is also impacted by an individual’s relative fighting aptitude, as this would impact contest outcomes [10]. For example, large, superior individuals are perhaps more prone to engaging in combat over a resource, particularly when competing with a smaller or less powerful individual [11]. In contrast, when rivals are relatively matched in size or quality, there is a higher probability that conflict may develop into aggressiveness.

Parasitoids are vital species in the suppression of insect pest populations. Many species of parasitoids are used for controlling insect pests, such as the genus *Anastatus* Motschulsky, 1859 (Hymenoptera: Eupelmidae). It is the most globally distributed genus of Eupelmidae, with over 135 known species. *Anastatus* has been reported as primary endoparasitoids, attacking more than 200 insect species—primarily of the order Blattodea, Hemiptera, Lepidoptera, Mantodea, Neuroptera, Orthoptera, and Phasmida—causing economic damage to crops [12]. *Anastatus* sp. has been used in China to suppress the litchi stink bug *Tessaratoma papillosa* Drury (Hemiptera: Pentatomidae) population with successful outcomes since the 1960s [13]. *Anastatus japonicus* Ashmead has been used to control the invasive brown marmorated stink bug, *Halyomorpha halys* Stål (Hemiptera: Pentatomidae) in forests and peach orchards, with observed parasitism rates of between 48.7 and 77.2% [14]. This species is present across the whole Palaearctic area, attacking more than 15 host species of the hemipteran and lepidopteran order [15]. It is also present in Canada and the United States (Nearctic region), and its existence in North America is thought to have resulted from invasions linked to biological control of the spongy moth, *Lymantria dispar* (L.) (Lepidoptera: Erebidae) [16].

Although male–male combats are well documented in parasitoids, only a few studies have been conducted with Hymenopteran parasitoid species in the context of mating behavior, particularly linked with precopulatory mate-guarding behavior [17,18,19]. Interestingly, the outcome of male–male combat among *Ibalia japonica* (Hymenoptera: Cynipoidea: Ibaliidae) members is attributed to the arrival order of males but not the body size [20], which shows species-specific characteristics of this phenomenon. As *A. japonicus* males can only survive for around 5 days while females survive eight times longer [12], male–male combats would be critical for either reproductive success or resource competition. Here, we examined factors affecting the frequency and intensity of aggressive interactions between *A. japonicus* males. The following hypotheses were tested: (1) the frequency and intensity of male contests will increase with increasing male density, as a function of encounter rate (e.g., [7]) and decreasing female density, which increases female value (e.g., [21]); (2) males can differentiate between virgin and mated females and will be more aggressive in the presence of the former given their greater value as mates; (3) males assess opponents such that fights are more frequent between asymmetrically sized males than between those of the same size; (4) fed males will be more aggressive than starved males because energy limitation will reduce fighting ability and the likelihood of success. In addition, an improved understanding of male aggressive interactions would help us gather more knowledge on the behavioral ecology of the egg parasitoid and ultimately design an efficient field release strategy for *A. japonicus* to manage hemipteran and lepidopteran pests.

## 2. Materials and Methods

### 2.1. Insect Colonies

*A. japonicus* parasitoids were sourced from a colony established in 2015 from parasitized *H. halys* eggs collected in a peach orchard in Beijing, China (N40°02′06″; E116°12′41″), which was maintained at MARA-CABI Joint Laboratory for Biosafety, Beijing, China [22]. The parasitoid colony was held in ventilated acrylic rearing cages (25 cm × 25 cm × 25 cm) in a climate-controlled chamber set to 22 ± 1 °C, 60 ± 5% RH, and 16:8 (L:D) photoperiod. Adult wasps were fed diluted 20% honey solution on cylindrical sponges, which were replaced twice weekly. The colony of *A. japonicus* was reared on factitious host frozen eggs of *Antheraea pernyi* (Guerin-Meneville) (Lepidoptera: Saturniidae) obtained from the Beneficial Insect Rearing Facility at the Institute of Plant Protection Research, Guangdong Academy of Agricultural Sciences, Guangzhou, Guangdong, China. A cluster of host eggs (<500) was pasted on a piece of paper (100 × 100 mm) with clear liquid glue (Pritt Liquid Glue, Henkei Industry and Trade Co., Ltd., Hangao, Guangdong, China) and presented for two weeks in the rearing cages for parasitization and then kept in the small glass cage (25 cm × 25 cm × 25 cm) until emergence. Male relatedness was lowered by obtaining several-generation males by separating the virgin grandmothers during oviposition. The virgin females lay unfertilized eggs as *A. japonicus* has a haplodiploid system of reproduction; thus, only male offspring would be produced. Emerging males from parasitized *A. pernyi* eggs were separately enclosed in vials with ventilated openings to reduce fighting before initiating the experiment. All wasps were less than 24 h old when used in experiments. All bioassays were replicated thirty times and were conducted in a climate-controlled chamber (BluePard Series, Yiheng Technology Company, Shanghai, China) under the above-described physical conditions.

### 2.2. Male–Male Aggression as a Function of Male Density

To explore whether the intensity of fighting is affected by the number of competitors, we confined only males in six densities: 1, 2, 4, 8, 15, and 32 males per container. Groups of virgin males (all <24 h post-emergence) were held in ventilated plastic cylinders (6 cm ht × 8 cm diam) for 24 h with 20% diluted honey provided on cotton wool. Surviving, dead, and injured males were then isolated in 1.5 mL polyethylene tubes and examined under a stereomicroscope (SZ2-ILST, Olympus Co., Tokyo, Japan) to assess visible injuries. Injuries were categorized on a scale of 0–5 (Table 1, e.g., the loss of antennae scored 0.5 points), drafted from Murray with some modifications [7], and a mean injury level tallied for each wasp.

### 2.3. Male–Male Aggression as a Function of Related Males

Males were categorized by surface fluorescent powder marking as sibs (related; sons of the same mother) and non-sibs (mixed; sons of unrelated mothers). The virgin male competitors of the above-mentioned groups of either 2 or 8 densities were placed into cylindrical arenas (as above) for 24 h, and deaths and/or injuries were scored in the same way as described in the first experiment.

### 2.4. Male Aggression as a Function of Body Size

Newly emerged males (<24 h) were selected randomly from the stock colony, isolated in 1.5 mL vials, and placed on ice for a few minutes (<3 min) until they became immobile. Immediately after immobilization, each wasp was removed using a fine camel hair brush, and its hind tibia (Ht) length was measured under a stereomicroscope. Virgin males were then divided into two groups, large (Ht > 0.06 mm) and small (Ht < 0.04 mm), based on the Ht length, and males with sizes between small and large were removed. Three different contest categories were then constructed in experimental arenas (as above): small vs. small, large vs. large, and large vs. small. A single virgin female (<24 h post-emergence) was presented in each arena to elicit male aggressive behavior and deaths and/or injuries were scored as described above.

### 2.5. Aggression as a Function of Male Condition (Fed vs. Unfed)

Newly emerged males (<24 h) were isolated in 1.5 mL vials and divided into two groups; one group was provided with a cotton wick of diluted honey (20%) and the other with water only. Both treatment groups were held separately in an incubator for three days before experimental trials. Trails were conducted by placing a pair of virgin males (honey-fed + starved) in ventilated cylinders (as above) for 24 h, with each pair in the presence of a virgin female (<24 h post-emergence). Male aggressive behavior in the form of deaths and/or injuries was scored in the same way as noted above.

### 2.6. Male–Male Aggression as a Function of Female Mating Status and Density

The effect of the presence of newly emerged (<24 h old) female *A. japonicus* on male fighting intensity was studied by altering the number of present females (0, 2, 4, 8, 15, and 20 females) with groups of four <24 h old virgin males. Females were divided into two groups, virgin and mated; thus, each of the number treatments had either all virgin or all mated. The experimental arena, procedure, and fighting intensity were as described previously. We also considered the mean severity of injury for this treatment from Table 1, which we categorized as either slight or severe (≥3 score: severely injured).

### 2.7. Statistical Analysis

All data analyses were carried out in SPSS software (v. 26). Datasets were checked for normality (Shapiro–Wilk) and homoscedasticity (Levene’s test) prior to analysis. The proportion of dead and injured wasps and the proportion of severity of injury were analyzed by the generalized linear model GLM Wald χ^2^ with binomial errors using a log link function. The proportional data in impact of competitor density on male–male combat was analyzed by generalized linear mixed model GLMMs F with a linear distribution and log link function. Injury scores with more than 2 groups were analyzed by the Kruskal–Wallis H or Mann–Whitney U test, where two groups were compared. Graphs were generated in Origin 2019.

## 3. Results

### 3.1. Impact of Competitor Density on Male–Male Combat

**Figure 1:**
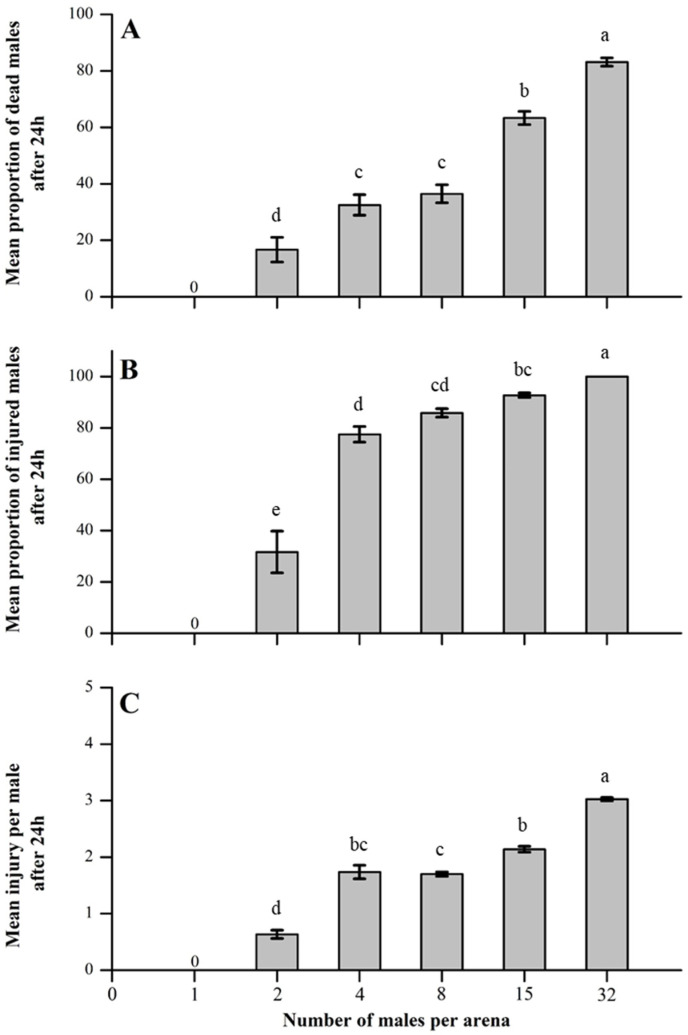
Impact of competitor density on male–male fighting intensity among *A. japonicus*. Fighting intensity was noted as the (**A**) mean proportion of dead males, (**B**) mean proportion of males injured, and (**C**) mean injury per male after 24 h. Error bars are used to express standard errors, and different letters on bars denote significant differences at *p* < 0.05.

Competitor group size had a significant effect on the mean proportions of dead and injured males and the mean injury per male. When *A. japonicus* males were allowed to interact in an arena, we found that the proportions of dead and injured males increased significantly as the group size of competitors increased (proportion of dead males, GLMMs F_4,145_ = 427.467, *p* < 0.001; proportion of injured males, GLMMs F_4,145_ = 3.341, *p* < 0.001). Similarly, the mean severity of injury recorded per male increased significantly as the group size of competitors increased (H = 120.053, df = 4, *p* < 0.0001) (Figure 1).

### 3.2. Impact of Related Males on Male–Male Combat

**Figure 2:**
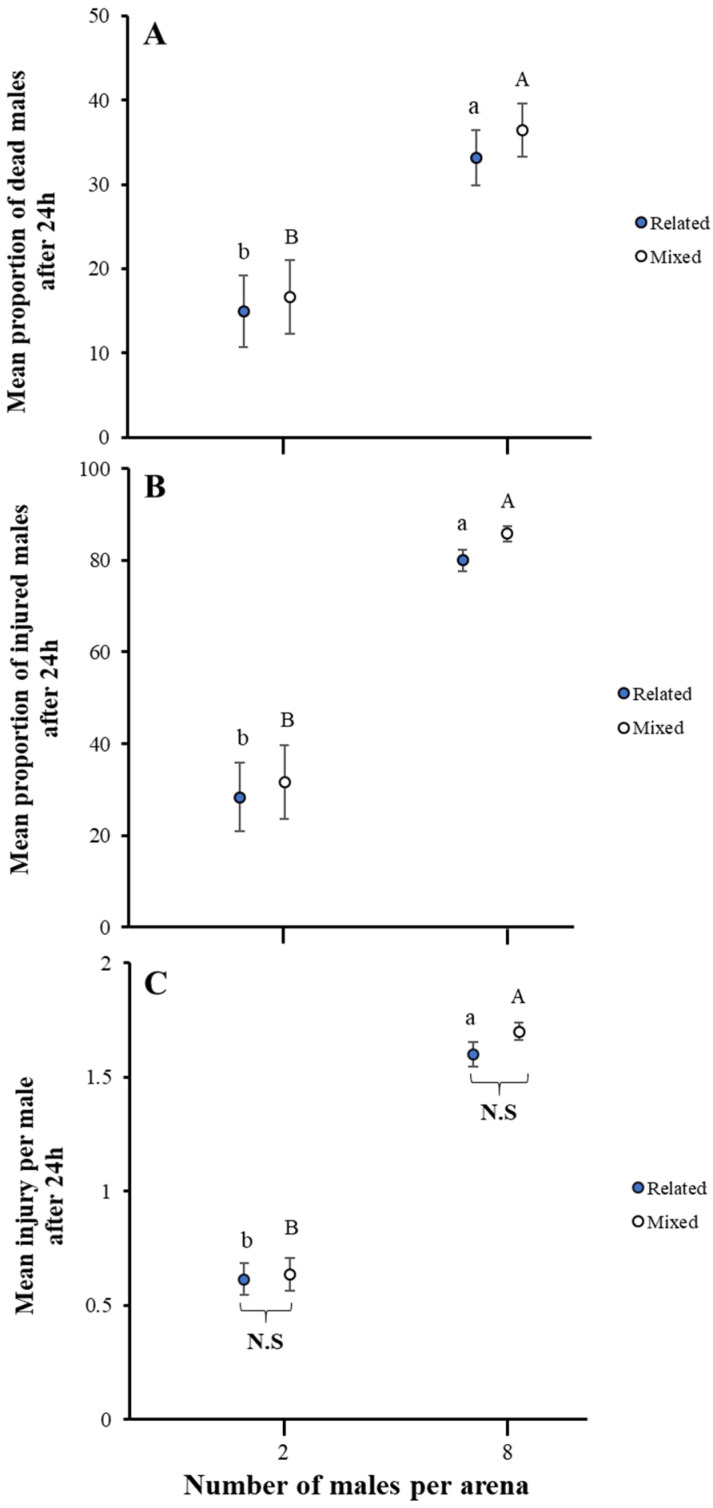
Impact of related and mixed males group size on fight intensity in *A. japonicus*. Fight intensity was noted as the (**A**) proportion of dead males, (**B**) proportion of males injured, and (**C**) mean injury per male after 24 h. Error bars are used to express standard errors, and different letters on bars denote significant differences at *p* < 0.05. Both relatedness and mixed factors have no significant differences and are marked as N.S only at the bottom.

When *A. japonicus* males were enabled to interact with related and mixed (non-related) males in an arena, we found that aggression increased significantly as the group size of competitors increased in mean injury (related males: U = 13.000, *p* < 0.05; mixed males: U = 6.000, *p* < 0.05), the proportion of male injured (related males: χ^2^ = 178.500, *p* < 0.05; mixed males: χ^2^ = 216.000 *p* < 0.05), and the proportion of dead males (related males: χ^2^ = 225.000, *p* < 0.05; mixed males: χ^2^ = 240.000, *p* < 0.05) (Figure 2). On the other side, the impact of similar group size on related males was non-significant on mean injury (two males: U = 448.500, *p* = 0.980; eight males: U = 372.000, *p* = 0.246), the proportion of males injured (two males: χ^2^ = 439.000, *p* = 0.849; eight males: χ^2^ = 345.000, *p* = 0.095), and the proportion of dead males (two males: χ^2^ = 435.000, *p* = 0.783; eight males: χ^2^ = 397.500, *p* = 0.426) (Figure 2).

### 3.3. Impact of Body Size on Male–Male Combat

**Figure 3:**
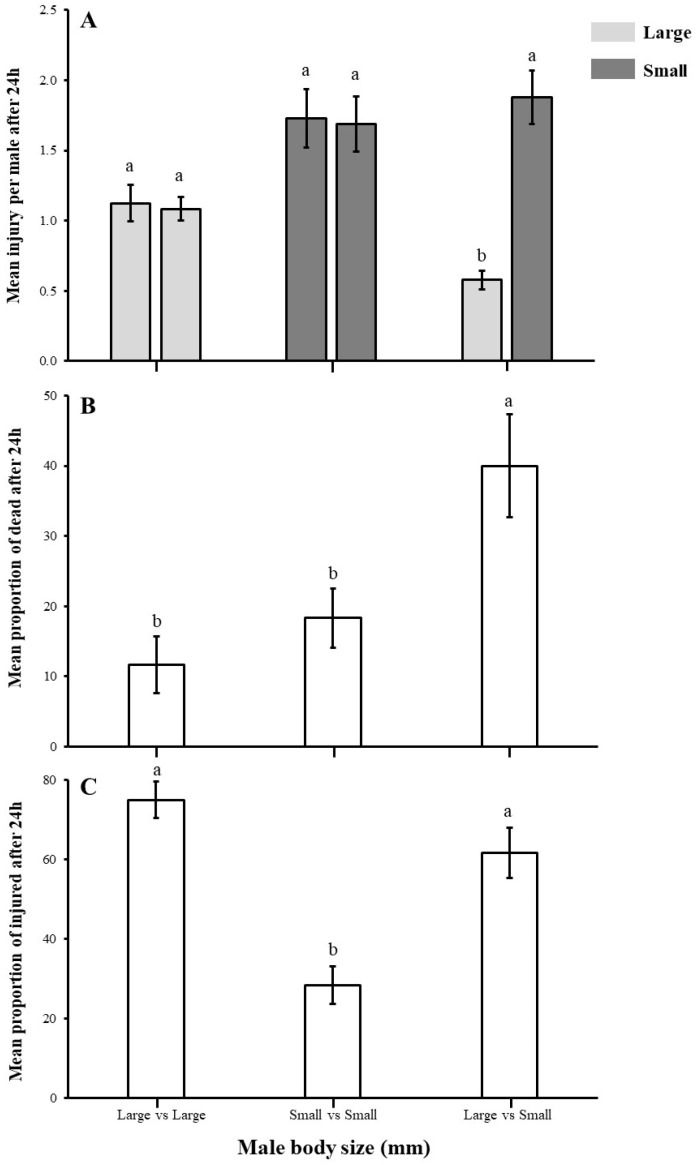
Body size influences fight intensity in *A. japonicus*. Fight intensity was measured as (**A**) mean injury per male (mean ± SE), (**B**) proportion (mean ± SE) of dead males, and (**C**) proportion of injured males (mean ± SE) in male–male combat in *A. japonicus* in arenas containing similar and opposite status (i.e., body size) of mates in one group. Different letters on the bars denote significant differences at *p* < 0.05; however, bars with same letter are non-significant.

The results demonstrate that the size of the male *A. japonicus* has a significant impact on the proportion of injuries and dead males; however, a non-significant impact on the mean injury was recorded in experimental males. The mean injury level of both males in large vs. large and small vs. small exhibited no significant differences, meaning that both males were injured during combat (U, large vs. large; U = 10.000, *p* = 0.194, small vs. small; U = 284.500, *p* = 0.940). However, males in small vs. small combat showed higher injury levels compared with males in large vs. large combat. Interestingly, in large vs. small male combats, larger males showed dominance over smaller males, and their mean injury was significantly lower than smaller ones (χ^2^ = 13.500, *p* < 0.05) (Figure 3A).

The smaller males die more often than the larger males. Larger insects hit smaller insects in combat, and the mortality of the smaller male parasitoids was higher compared with larger males. Death proportion was significantly higher in large vs. small combat when compared to large vs. large and small vs. small treatments (χ^2^ = 15.225, df = 2, *p* < 0.05) (Figure 3B).

The proportion of injured males was higher in the combats with larger males and was significantly higher than in small vs. small insect combat. Moreover, male parasitoids in large vs. large contests were injured more compared with large vs. small insect combat (χ^2^ = 44.203, df = 2, *p* < 0.05) (Figure 3C).

### 3.4. Impact of Starved and Honey-Fed Males on Male–Male Combat

**Figure 4:**
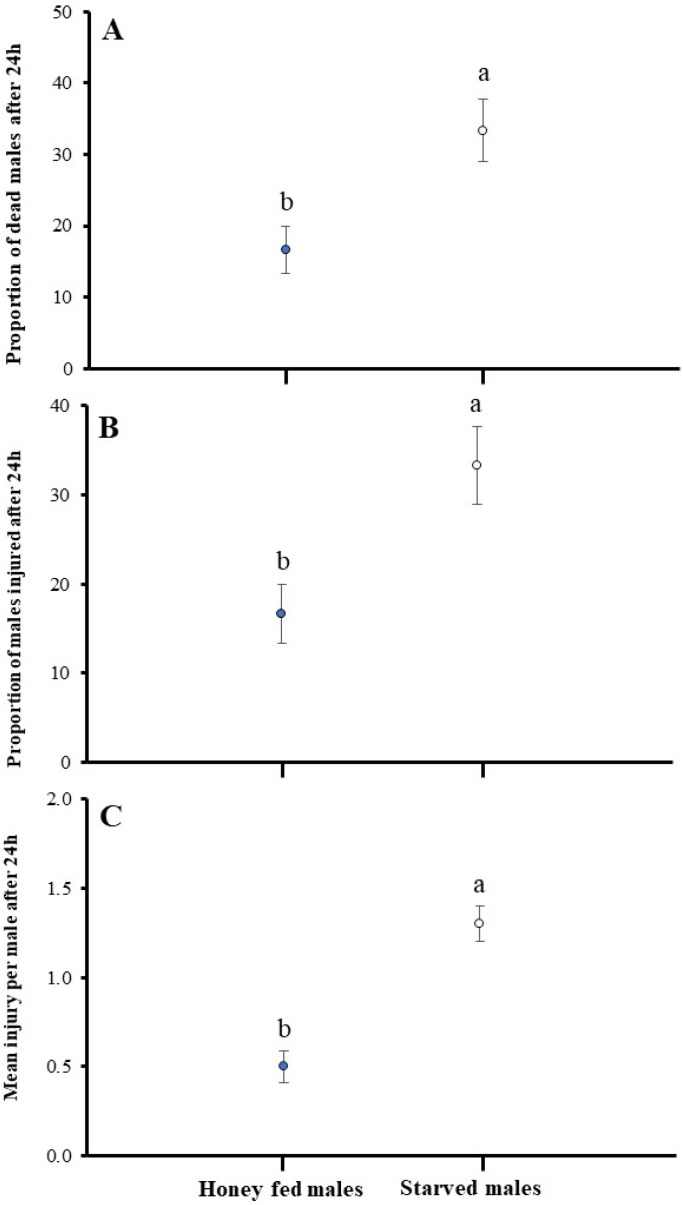
Impact of honey-fed and starved males on fight intensity in *A. japonicus*. Fight intensity was measured as the (**A**) proportion of dead males, (**B**) proportion of males injured, and (**C**) mean injury per male after 24 h. Error bars are used to express standard errors, and different letters on bars denote significant differences at *p* < 0.05.

The results demonstrate that the honey-fed males show dominance over starved ones and have a significant impact in combat on mean injury per male (U = 17.70, *p* < 0.05), the proportion of dead (U = 125.670, df = 1, *p* < 0.05), and the injured male proportion (U = 59.43, df = 1, *p* < 0.05) (Figure 4).

### 3.5. Impact of Female Presence on Male–Male Combat

**Figure 5:**
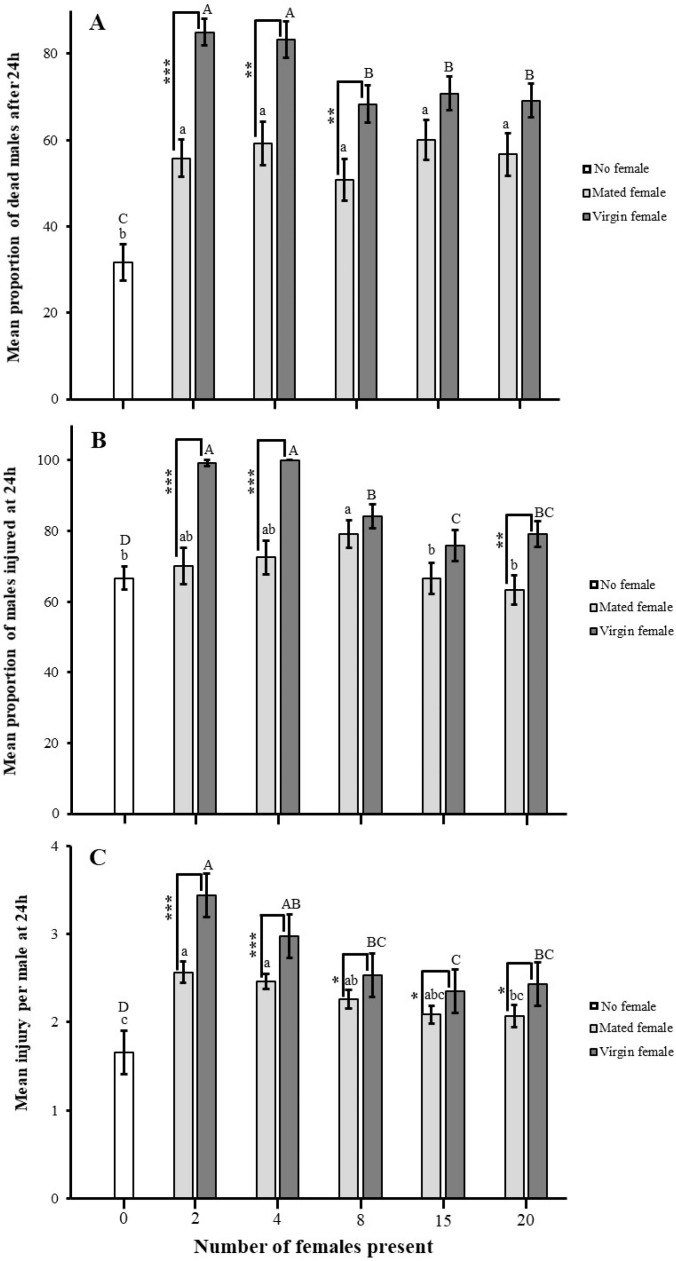
Impact of female presence on fight intensity in *A. japonicus*. Fight intensity was measured as the (**A**) proportion of dead males, (**B**) proportion of males injured, and (**C**) mean injury per male after 24 h. Error bars are used to express standard errors, and different small letters on bars denote significant differences at *p* < 0.05 among zero females and mated females; similarly, capital letters represent zero females and virgin females. Asterisk indicates significant differences among virgin and mated females at * *p* < 0.05, ** *p* < 0.001, and *** *p* < 0.0001 for χ^2^ test.

The presence of females significantly increased male aggression, as measured by the proportions of dead (no female (NF) vs. mated female (MF): χ^2^ = 27.126, df = 5, *p* < 0.05, and NF vs. virgin female (VF): χ^2^= 122.135, df = 5, *p* < 0.05) and injured males (NF vs. MF: χ^2^= 8.753, df = 5, *p* > 0.05, and NF vs. VF: χ^2^= 99.982, df = 5, *p* < 0.05) and the mean injury per male (NF vs. MF: H = 38.639, df = 5, *p* < 0.05 NF vs. VF: H = 86.306, df = 5, *p* < 0.05). The lowest number of females showed the highest proportion of dead males in the presence of both virgin and mated females. However, the rest of the female numbers did not significantly influence the proportion of dead males in mated females. In contrast, virgin females have a significant impact on the proportion of dead males. (Figure 5A). Similar results were found for injured males (Figure 5B) and mean injury per male (Figure 5C).

**Figure 6:**
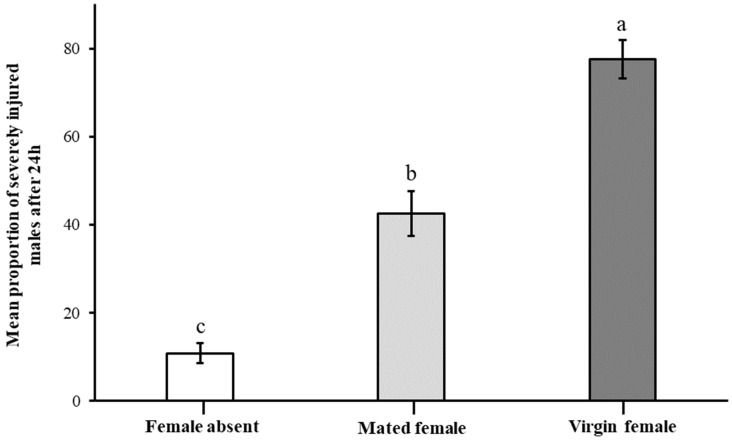
Proportions of males severely injured after combat for 24 h under conditions of no female, mated female, and virgin female. Severe injury generally involves the loss of two legs and whole antennae or even more serious injuries. The different letters indicate significant differences. The error bars indicate means ± standard errors and different letters on bars denote significant differences at *p* < 0.05.

Comparing the males in the treatment with no females, including virgin and mated females, males presented with a virgin female significantly increased their fighting intensity (proportions of dead: χ^2^= 103.304, df = 5, *p* < 0.05; and injured proportion: χ^2^= 99.982, df = 5, *p* < 0.05 males; and mean injury per male: H = 86.375, df = 5, *p* < 0.05), and they were also measured as severely injured with the loss of the whole antennae and more than one leg (H = 19.500, df = 2, *p* < 0.05) (Figure 6).

## 4. Discussion

Our findings validate theoretical speculation about how aggression in male *A. japonicus* can be influenced by a competitive setting. Particularly, higher male density resulted in more fights, ultimately less courting, and increased male mortality. Within contests, larger competitors fared considerably better than smaller ones. Similarly, honey-fed males were dominant over starved males. Fighting occurred more frequently when there was an inequality in fighting abilities between opponents, which proposed that self-ability shapes contest behavior.

Variations in male–male interaction and density can be affected in two ways, leading and influencing fighting behavior in opposite directions [23]. First, a higher male density will result in a higher encounter rate among individuals. This is expected to increase fighting, which has been studied in the fig wasp *P. pilosa* [7]. Our results correspond to fighting intensity, and we noticed an equivalent pattern. Second, male density changes the cost of fighting, and it is expected to influence fight intensity as well [24]. More specifically, increased competitor density decreases the benefit obtained by fighting because (1) fighting is inefficient because other males would be mating with females simultaneously [7] and (2) the possibility of beating all competitors is lowered with the time spent on fighting [23]. Murray (1987) explained the interaction among these opposing elements; for example, the overall greatest fighting cost is observed at median male densities: fighting becomes frequent at low densities and fiercer at high densities [7]. Nonetheless, given the small number of males used per treatment, we would expect a positive relationship between the density and cost of fighting in this orbit. Greater male densities also lead to a reduction in the possibility of starting a fight [23], but our findings suggest that fighting always occurs.

Male–male combats among animals are influenced by different environmental factors such as varying temperatures, as *A. japonicus* is very sensitive to high temperatures, which affects its mobility (M. Y. Ali, unpublished data). An earlier male–male combat study on *A. disparis* (Syn. *A. japonicus*) was carried out at a temperature of 28 °C [25]. However, our experience with *A. japonicus* revealed that this temperature is harsh for the survival of male wasps. Therefore, we assume that the dead ones in Liu et al.’s (2017) study could potentially be due to extreme temperatures and not fighting [25]. Furthermore, the higher fighting intensity in our case is in contrast with *A. disparis* possibility due to the fact that we provided 24 h, whereas Liu et al. (2017) [25] provided only 3 h for the experiment. However, any predictions of the impacts that our results could have on the field population of male *A. japonicus* must be interpreted with caution, as male parasitoid encounters may not happen often in the field.

Hamilton’s theory of kin selection proposes that fight intensity should be lower for closely related rivals than for less related ones, and greater altruism should be evident in the former [26]. However, when *A. japonicus* males were allowed to interact with both non-relatives and relatives in an arena, we found no evidence that male competitors adjust their fighting behavior in response to relatedness, so our findings reject Hamilton’s theory. This is potentially due to the fact that *A. japonicus* may be unable to discriminate kin (i.e., phenotypic kin recognition), an ability that is rarely observed in nonsocial insect species [8,27,28]. In contrast, males with common early social experience may treat each other as kin or even not real relatives, resulting in less aggression following the prediction of kin selection theory [29]. On the other hand, the intensity of male fighting was greater under female presence, which is likely due to mating opportunities [30]. In addition, the fighting was significantly higher in combat with virgin females because of mating opportunities as *A. japonicus* female exhibits monandry behavior (M.Y. Ali, unpublished data), so the potential benefits of winning can exceed the costs of fatal combat. However, increasing female numbers in the arena did not significantly reduce fighting intensity among males. Yin et al. (2023) also studied the fighting behavior of *A. disparis* with high, intermediate, and low relatedness; however, a relatedness and no relatedness study was still missing, and our results express that these variables are of great value [31].

*Anastatus japonicus* females mate only once, and, after mating, the female parasitoids alter their foraging behavior to host-seeking. According to a field study of the braconid aphid parasitoids, mated *Aphidius nigripes* and *A. ervi* females attracted significantly fewer males than virgin females [32]. This is possibly due to the presence of some unique pheromones only emitted by virgin females that cause extreme fights. Secretions transferred during male ejaculation switch off pheromone production in females, a phenomenon that is known as pheromonostasis [33]. It has even been found that in *Heliconius* butterflies, males transfer “anti-aphrodisiacs” pheromones to females during mating, reducing the attraction of females to following males and ultimately less fighting [34]. Furthermore, aggressive/fighting intensity is higher in our study, in contrast with Liu and Hao (2019) [35], possibly because they used 2-day-old virgin females in their experiments with *A. disparis*, which follows our hypothesis that newly emerged virgin females produced higher amount of sex pheromones, ultimately attracting more males and leading to extreme male–male combat (M. Y. Ali, unpublished data). Although significant differences in male aggression to unmated and mated females were noted, they both appeared to increase fighting in males compared with no females in the arena.

Precopulatory mate-guarding behavior has been found in several hymenopteran parasitoids [20]. Although it is not yet confirmed, this mate-guarding behavior might also be used by *A. japonicus* males to guard over the egg mass before females have emerged and then mate females that subsequently emerge. The likelihood of male–male combat would also be impacted by the sex ratios of egg masses when sibling males are simultaneously emerging and waiting for the emergence of virgin females. Moreover, combat would also be influenced by the proportion of sibling mating (likely occurring on the emerged egg mass) versus non-sibling mating (dispersal then mate). Further studies are needed to investigate mate-guarding behavior and related male–male combats under these varied mating scenarios.

Body size and self-fighting ability are among the most crucial life-history traits of organisms [36] and are generally positively linked with many fitness elements in parasitoids [37,38]. Specifically, we may anticipate that smaller size males would refrain from fighting or remain recessive in combat because being larger greatly increases competitive success [39]. However, our study suggests that *A. japonicus* males always fight and that fighting between two males starts right after they face each other. According to the perspective of Enquist and Leimar (1990) [24], all conflicts should be settled by fighting rather than giving up if the value of the resource being contested is much more than the value of prospective future resources. In these circumstances, it is not in the interests of weaker rivals to give up a fight: even if the chances of victory are extremely low. A similar case happened in *A. japonicus*; there is only one chance of winning mating, and this resource is highly valuable. In other words, males fight as they have a lot to gain and not much to lose (desperado effect) [40]. It is very possible that the greater quality male makes sure he can kill the lower quality opponent (dominance hierarchy; [41]) to gain resource value (i.e., mating). However, body size is not an important factor in male–male combat of I. japonica, which might be attributed to the fact that body size variation in male koinobiont parasitoids is very small [19,20].

Our study also explains why the energetic (honey-fed) male shows dominance over starved ones. Fed wasps have a greater amount of polar metabolites linked with the rapid mobilization of energy (e.g., glucose, glycerol, glutamate, trehalose,) than starved wasps [42]. This results in enhanced resource-holding potentials when contest interactions are energetically demanding. Tsai et al. (2014) also found that 4-day-old honey-fed males of *Nasonia vitripennis* enhanced their contest success; however, there was no effect on the younger ones (1 d old), which is probably because newly emerged wasps already have some energy reserves from the host [43]. The outcome of another dietary supplementation study reveals that males fed with honey water show significantly higher fighting intensity than unfed fighting-experienced males [44].

In summary, our study shows how different biological factors affect lethal male–male combat in the parasitoid *A. japonicus*. Fighting among males always occurs; however, the intensity of contests can be affected by several factors such as those presented in our study. The highest intensity of fighting was seen with combat involving females, and the aggression of males was further increased when provided with virgin females. Further studies are needed to evaluate the phenomenon with the possible involvement of sex pheromones, which males might use to differentiate between virgin and mated females.

## Figures and Tables

**Table 1:** Criteria used for scoring the severity of injuries on male *A. japonicus*.

Description of Injury *	Score for Each Injury
Loss of Part or Whole Antenna	0.5
Loss of Part or Whole Tarsi	1.0
Loss of Part or Whole Tibia (Plus Tarsi)	2.0
Loss of Part or Whole Femur (Plus Tibia and Tarsi) and Bruise	3.0
Loss of Part or Whole Coxa (Plus Femur, Tibia and Tarsi)	4.0
~<Half Severed Abdomen or Head, No Evisceration	5.0

* The injury was scored based on damage to one or both appendages and equated.

## Data Availability

The data are available from the corresponding author upon reasonable request.
